# 3,5-Diiodo-L-Thyronine (T2) Administration Affects Visceral Adipose Tissue Inflammatory State in Rats Receiving Long-Lasting High-Fat Diet

**DOI:** 10.3389/fendo.2021.703170

**Published:** 2021-07-12

**Authors:** Giuseppe Petito, Federica Cioffi, Elena Silvestri, Rita De Matteis, Davide Lattanzi, Pieter de Lange, Assunta Lombardi, Maria Moreno, Fernando Goglia, Antonia Lanni, Rosalba Senese

**Affiliations:** ^1^ Department of Environmental, Biological and Pharmaceutical Sciences and Technologies, University of Campania “L. Vanvitelli”, Caserta, Italy; ^2^ Department of Sciences and Technologies, University of Sannio, Benevento, Italy; ^3^ Department of Biomolecular Sciences, Urbino University, Urbino, Italy; ^4^ Department of Biology, University of Naples Federico II, Naples, Italy

**Keywords:** visceral white adipose tissue, inflammation, hypoxia, microRNA, angiogenesis

## Abstract

3,5-diiodo-thyronine (T2), an endogenous metabolite of thyroid hormones, exerts beneficial metabolic effects. When administered to overweight rats receiving a high fat diet (HFD), it significantly reduces body fat accumulation, which is a risk factor for the development of an inflammatory state and of related metabolic diseases. In the present study, we focused our attention on T2 actions aimed at improving the adverse effects of long-lasting HFD such as the adipocyte inflammatory response. For this purpose, three groups of rats were used throughout: i) receiving a standard diet for 14 weeks; ii) receiving a HFD for 14 weeks, and iii) receiving a HFD for 14 weeks with a simultaneous daily injection of T2 for the last 4 weeks. The results showed that T2 administration ameliorated the expression profiles of pro- and anti-inflammatory cytokines, reduced macrophage infiltration in white adipose tissue, influenced their polarization and reduced lymphocytes recruitment. Moreover, T2 improved the expression of hypoxia markers, all altered in HFD rats, and reduced angiogenesis by decreasing the pro-angiogenic miR126 expression. Additionally, T2 reduced the oxidative damage of DNA, known to be associated to the inflammatory status. This study demonstrates that T2 is able to counteract some adverse effects caused by a long-lasting HFD and to produce beneficial effects on inflammation. Irisin and SIRT1 pathway may represent a mechanism underlying the above described effects.

## Introduction

Nowadays, metabolic syndrome/obesity are health problems of epidemic proportions, which are often the cause of related pathologies such as type 2 diabetes, hypertension, and cardiovascular disease. Metabolic disorders are multifactorial pathologies with a close relationship between nutrient excess and activation of the innate immune system in most organs involved in energy homeostasis (1–3). Some studies have also shown that inflammation is another element of the metabolic disorder associated with the excessive visceral fat accumulation (4–6). Indeed, increased adipose mass is associated with tissue modifications (7) such as: a) a low-grade inflammatory state due to the infiltration of the mediators of the immune response (monocytes/macrophages and lymphocytes) (8, 9); b) pro-inflammatory cytokine recruitment [e.g., Tumor Necrosis Factor A (TNF-A), Interleukin 6 (IL-6), Plasminogen Activator Inhibitor-1 (PAI-1) and Monocyte Chemoattractant Protein-1 (MCP-1)] (10, 11) and c) occurrence of hypoxia due to hampered perfusion of the enlarged adipocytes or increased oxygen consumption (12).

Hypertrophic adipocytes are much more susceptible to the infiltration of macrophages and to cell death than normal adipocytes, and increased number of tissue macrophages causes an increased secretion of inflammatory cytokines (13). Adipose tissue activated macrophages (ATMs) are polarized according to changes in their environment, forming different macrophage phenotypes: M1 macrophages and M2 macrophages (14). M1 phenotype is mainly involved in pro-inflammatory responses (9, 15, 16) and M2 phenotype is mainly involved in anti-inflammatory responses and in the maintenance of tissue homeostasis (17). During the progression of the high fat diet-induced obesity, in white adipose tissue the increased M1/M2 ratio appear to be a major contributing factor to the chronic inflammation (18). Indeed, improving the inflammatory environment by modulating macrophages polarization is an effective method for the treatment of diseases.

In adipose tissue, hypoxia is an early upstream initiator for adipose tissue dysfunction (19), and may initiate inflammation by inducing the hypoxia inducible factor 1A (HIF-1A) regulated gene program (20). In addition, the stimulation of an unhealthy angiogenesis leads to an increased nutrient intake into the adipocytes and recruitment of inflammation mediators at the tissue level. In this way, a vicious circle is established in which the activation of angiogenesis determines a further increase in adipocyte volume and, subsequently, an increase in the inflammatory state of adipose tissue (21). Many molecules have been implicated as positive regulators of angiogenesis, among them vascular endothelial growth factor (VEGF) is a pivotal angiogenic factor (22). Recent evidences are highlighting the involvement of miRNAs in the regulation of angiogenic factors. In fact, it has been shown that miR126 targets VEGF, Sprouty-related EVH1 domain-containing protein 1 (SPRED1) and phosphoinositol-3 kinase regulatory subunit 2 (PIK3R2) that play a fundamental role in endothelial cell signaling or in vascular function (23, 24). In particular, SPRED1 is a member of the SPROUTY/SPRED family of proteins that act as negative regulators of RAS-RAF interaction and mitogen-activated protein kinase (MAPK) signaling (25, 26). PIK3R2, instead, negatively regulates the activity of phosphoinositide 3-kinase (PI3K) (27).

The study of hexogen/endogen agents that might affect these processes could be important to better understand the underlying mechanisms and to try to identify factors capable of countering the related dysfunctions. Among these agents, 3,5-diiodo-L-thyronine (T2), an endogenous metabolite of thyroid hormones, is attracting growing interest. Indirect *in vivo* evidence indicates that T2 is formed from 3,5,3’-triiodothyronine (T3) through deiodination (28–31), although conversion of T3 to T2 has not been demonstrated *in vitro*. Serum concentration of T2 has been reported to be within the picomolar range, even if its levels were difficult to detect (32, 33).

In the last decades many laboratories have demonstrated that T2 exhibits interesting favorable effects in different metabolically active tissues such as liver (34–39), skeletal muscle (40, 41), heart (42), and adipose tissues (43, 44). In male wistar rats housed at thermoneutrality and fed on a high fat diet, simultaneous T2 administration reduces hepatic fat accumulation and hyperlipidemia (34), and induces a protein profile favoring a shift toward type 2 skeletal muscle fibers (40). Padron et al. reported that T2 administration at different doses to aging rats reduced body- and retroperitoneal fat mass gain, ameliorated glucose tolerance, did not alter heart mass and heart rate, and only lowered serum 3,5,3’,5’-tetraiodothyronine (T4) and T3 levels at the two high doses (45). In addition, T2 may preserve liver mitochondrial integrity by inducing protective repair mechanisms against mtDNA oxidative stress (46). Lietzow J. et al. have recently confirmed the beneficial hepatic fat reduction by T2 and induction of fatty acid oxidation in mice fed a HFD (39). Moreover, we have recently shown that, in white adipose tissue, T2 induces adipocyte browning (44), affects adipocyte morphology (already measurable after 2 weeks and persistent at 4 weeks of treatment) and tissue vascularization (47).

Most of these studies were performed in animals receiving a HFD for four weeks with a simultaneous administration of T2. Based on these considerations, the aim of this study was to investigate whether T2 might reverse the inflammatory state after its induction through a long-lasting HFD (14 weeks). We focused our attention on the putative effects of T2 on: i) the modulation of M1 and M2 macrophages phenotype; ii) the main anti- and pro-inflammatory adipocytokines/agents, iii) the hypoxic environment potentially induced by the HFD; iv) regulation of angiogenesis factors, and v) the oxidative stress-induced DNA damage occurring in overweight animals.

## Materials and Methods

### Materials

General reagents were of the highest available grade and were obtained from Sigma Chemical (St. Louis, MO, USA). 3,5-diiodo-L-thyronine used for rats treatment was obteined from Sigma-Aldrich (St.Louis, MO, USA). Anti IL-6, anti TNF-A, anti PAI-1, and anti SIRT-1 antibodies were obtained from Abcam (Cambridge, UK). Anti IL-1RA, anti HIF-1A and anti VEGF antibodies were obtained from Santa cruz biotechnology (Dallas, TX, USA). Anti Phospho Akt (Ser473), Anti Phospho AMPK, Anti AMPK-Tot, Anti Akt (pan) and Anti IRS1 were obtained from Cell Signaling (Danvers, MA,USA). Anti P-IRS1 (Tyr612) was obtained from Invitrogen (Carlsbad, CA, USA). Anti B-actin antibody was obtained from Sigma Aldrich (St. Louis, MO, USA). Secondary antibodies, peroxidase anti-rabbit IgG and peroxidase anti-mouse IgG were obtained from Vector Laboratories (Burlingame, CA, USA). Adiponectin serum levels were determined using an Enzyme-Linked Immunosorbent Assay- ELISA kit (Abcam, Cambridge, UK). To detect TNF-A, IL-6, VEGF-A serum levels we used an Enzyme-Linked Immunosorbent Assay- ELISA kit, obtained from Invitrogen (Carlsbad, CA, USA). Irisin serum levels were measured using a Rat Irisin ELISA Kit obtained from MyBioSource (San Diego, CA, USA). DNA/RNA Oxidative Damage ELISA kit to measure oxidized guanine species from DNA was obtained from Cayman Chemical Company (Ann Arbor, MI, USA). TSH serum levels were measured using a specific enzyme linked immunosorbent assay (ELISA)-ELISA kit (Abnova, TWN, China). Free T4 and Free T3 serum levels were measured using the ELISA kit (Diametra s.r.l., Perugia, Italy). Serum levels of cholesterol were measured using a colorimetric enzymatic method employing a commercial kit (SGM Italia, Rome, Italy). QIAzol lysis buffer and miRNeasy micro kit were obtained from Qiagen (Hilden, Germany). To synthesize cDNA strands from RNA we used the SuperScript IV reverse Transcriptase for RT-PCR obtained from Invitrogen (Carlsbad, CA, USA). IQ SYBR Green supermix was obtained from Bio-Rad (Hercules, CA, USA). TaqMan^®^ miRNA assays for miR126 and U6B were obtained from Applied Biosystems (Foster City, CA, USA).

### Animals and Animal Care

Male Wistar rats (rattus norvegicus, 250-300g, aged eight weeks) were maintained one per cage in a temperature-controlled room at 28°C (thermoneutrality for rats) under a 12 hr dark/light cycle and housed in groups of five to six with unlimited access to water. All animals received humane care according to the criteria outlined in the Guide for the Care and Use of Laboratory Animals prepared by the National Academy of Sciences and published by the National Institutes of Health. All animal protocols were approved by the Committee on the Ethics of Animal Experiments of the University of Campania “L.Vanvitelli” (Italy) and the Italian Minister of Health (Permit Number: 704/2016-PR of the 15/07/2016; Project Number: 83700.1 of the 03/05/2015). Every effort was made to minimize animal pain and suffering. At the start of the study (day 0), and after seven days of acclimatization to thermoneutrality, the rats were divided into three groups. Each group was of five animals. The minimum sample size (n = 5) was calculated using a G* Power Test, developed by the University of Dusseldorf (http://www.gpower.hhu.de/). The power was 0.90, effect size (f) 1.2249 and the α was set at 0.05.

The three groups were treated as follows:

-The first group (N) received a standard diet ad libitum (total metabolizable percentage of energy: 60.4 carbohydrates, 29 proteins, 10.6 fat J -1; 15.88 kJ gross energy g-1; (Muscedola, Milan, Italy) for fourteen weeks with a daily intraperitoneal injection of vehicle (saline) for the last four weeks;
-The second group (HFD) received a high fat diet ad libitum [280 g diet supplemented with 395 g of lyophilized lamb meat (Liomellin, Milan, Italy), 120 g cellulose (Sigma-Aldrich, St. Louis, MO, USA), 20 g mineral mix (ICN Biomedical, Solon, OH, USA), 7 g vitamin mix (ICN), and 200 g low-salt butter (Lurpak, Denmark)]; (total metabolizable percentage of energy: 21 carbohydrates, 29 proteins, 50 fat J J-1; 19.85 kJ gross energy g-1) for fourteen weeks with a daily injection intraperitoneally of vehicle (saline) for the last four weeks. HFD animals were used as reference model of high-fat diet associated inflammation. Standard diet rats were only used as physiological control;
-The third group (HFD-T2) received the above-described HFD for 10 weeks and was subsequently treated for four weeks simultaneously with HFD and daily intraperitoneal injection of T2 (50 µg/100 g BW). The pharmacological dose of 50 μg T2/100 g BW was chosen after consideration of data from studies in which T2 was used. In fact, chronic treatment (4 wk) of rats with doses of 50 μg T2/100 g BW to rats pre-fed with a HFD for ten weeks, did not result in any thyrotoxic effect that might be of clinical relevance (44).

At the end of the treatment, the rats were anesthetized using an intraperitoneal injection of chloral hydrate (40 mg 100g-1 BW) and decapitated. Blood was collected and centrifuged at 2000 xg to collect plasma serum, which were divided into aliquots and stored at -20°C; mesenteric visceral white adipose tissues (vWAT) were excised, weighed and immediately frozen in liquid nitrogen and stored at -80°C for later processing.

### Measurements of Metabolic Parameters

Oxygen consumption (VO2) and carbon dioxide production (CO2) measurements were made using a four-chamber, indirect open-circuit calorimeter (Columbus Instrument), with one rat per chamber at a room temperature of 28°C to evaluate metabolic parameters, and testing rat from different groups simultaneously. Measurements were performed between 1100 and 1600 h. After a 1-h period of adaptation to the metabolic chamber, VO2 and VCO2 were measured. The system settings included a flow rate of 2 L/min, a sample line-purge time of 1,5 min., and a measurement period of 30 s. Rats were placed in separate 11-L calorimetry chamber with ad libitum access to water. Resting metabolic rates were calculated as the mean consecutive measurements during which the rats were not moving.

### Insulin Tolerance Test

For the insulin tolerance test, rats fasted for 5 h and were injected intraperitoneally with insulin (homolog rapid acting, 10 units/kg body wt in sterile saline; Novartis, Basel, Switzerland). Samples of blood were collected before the insulin tolerance test and at various times afterward (as indicated in the figures), and glucose values were determined by means of a glucose monitor (BRIO, Ascensia, NY, USA), calibrated for use with rats.

### Determination of Thyroid-Stimulating Hormone (TSH), Free 3,5,3’-Triiiodo-L-Thyronine (T3), Free 3,5,3’,5’-Tetraiodo-L-Thyronine (T4) Serum Levels

TSH serum levels were measured using a specific enzyme linked immunosorbent assay (ELISA) [TSH (Rodent) ELISA kit (Abnova, TWN, China)], according to the manufacturer’s protocol. Free T4 serum levels and free T3 serum levels were measured using the ELISA kit (Diametra s.r.l., PG, Italy).

### Determination of Adiponectin, Tumor Necrosis Factor Alpha (TNF-A), Interleukin 6 (IL-6), Vascular Endothelial Growth Factor A (VEGF-A) and Irisin Serum Levels

Adiponectin serum levels were determined using an Enzyme-Linked Immunosorbent Assay- ELISA kit (Abcam, CA, UK). TNF-A, IL-6, VEGF-A serum levels were measured using an Enzyme-Linked Immunosorbent Assay- ELISA kits, obtained from Invitrogen (Carlsbad, CA, USA). Irisin serum levels were measured using a Rat Irisin ELISA Kit obtained from MyBioSource (San Diego, CA, USA).

### Determination of Cholesterol Serum Levels and Glycaemia

Serum levels of cholesterol were measured using a colorimetric enzymatic method employing a commercial kit (SGM Italia, Rome, Italy). To measure glycaemia, rats were fasted overnight and glucose was determined by means of a glucose monitor (BRIO, Ascensia, NY), calibrated for use with rats.

### Histological Analysis and Adipocyte Size Determination

Samples of visceral fat pads were fixed by immersion in 4% (v/v) formaldehyde in 0.1M phosphate buffer (overnight, 4°C). The samples were dehydrated in ethanol, cleared, and embedded in paraffin blocks. The tissues were cut into serial 6-µm-thick sections and stained with hematoxylin-eosin for morphological examination. For adipocyte size quantification, evaluations were performed on 3 different hematoxylin-eosin slides (sections every 400 µm) for each animal and at least 400 adipocytes per animal were analyzed. Sections were viewed with a Nikon Eclipse 80i light microscope (Nikon instruments, Milan, Italy) at 20x magnification. Images were obtained with a Sony DS-5M camera connected to an ACT-2U image analyzer. The mean surface area and the frequency distribution were calculated from at least 4 rats for each group, adipocyte size distribution is presented as percentage of the total amount of cells.

### Preparation of Total Lysates

Tissue samples of vWAT were homogenized in Lysis Buffer containing 20 mM Tris-HCl (pH 7.5), 150 mM NaCl, 1 mM EDTA, 1 mM EGTA, 2.5 mM Na2H2P2O7, 1 mM b-CH3H7O6PNa2, 1 mM Na3VO4, 1 mM PMSF, 1 mg/mL leupeptin, and 1% (v/v) Triton X-100 (Sigma-Aldrich, St. Louis, MO, USA) using an UltraTurrax homogenizer, and then centrifuged at 16,000× g in a Beckman Optima TLX Ultracentrifuge (Beckman Coulter S.p.A., Milan, Italy) for 15 min at 4°C. The supernatants were then ultracentrifuged at 40,000 x RPM in a Beckman Optima TLX ultracentrifuge for 20 min at 4°C.The protein concentrations of the supernatants of the centrifuged lysates were determined using Bio Rad’s DC method (Bio Rad Laboratories, s.r.l., Segrate, Italy).

### Western Blot Analysis

Total lysates containing 30 µg protein were loaded in each lane and were electrophoresed on SDS-PAGE gels and transferred to nitrocellulose membrane and membranes were blocked with 5% (w/v) nonfat dry milk (in TBS-T). Primary antibodies were diluted in TBS with 0.01% (v/v) Tween 20 (TBS-T) and 5% (w/v) bovine serum albumin, while secondary antibodies were diluted in TBS with 0,01% (v/v) Tween 20 (TBS-T) and 5% (w/v) nonfat dry milk. Membranes were probed with the following antibodies: polyclonal anti IL-6 (Abcam- 1:1000 dilution), monoclonal anti TNF-A (Abcam- 1:200 dilution), polyclonal anti IL-1RA (Santa Cruz Biotechnology- 1:500 dilution), polyclonal anti-PAI-1 (abcam-1:000 dilution), anti HIF-1A (Santa Cruz Biotechnology- dilution 1:500), anti VEGF (Santa Cruz Biotechnology- dilution 1:500), polyclonal anti SIRT-1 (Abcam- 1:1000 dilution), Anti Phospho Akt (Ser473) (Cell Signaling- 1:1000 dilution), polyclonal Anti Phospho AMPK (Cell Signaling-1:1000 dilution), polyclonal Anti AMPK-Tot (Cell Signaling-1:1000 dilution), Anti Akt (pan) (Cell Signaling- 1:1000 dilution), monoclonal Anti IRS1 (Cell Signaling- 1:500 dilution), monoclonal Anti P-IRS1 (Tyr612) (Invitrogen- 1:1000 dilution) and monoclonal B-actin (Sigma Aldrich-dilution 1:1000). As secondary antibodies, peroxidase anti-rabbit IgG (Vector Laboratories-1:4000 dilution), peroxidase anti-mouse IgG (Vector Laboratories-1:4000 dilution) were used. Horseradish peroxidase-conjugated secondary antibodies were used for signal detection by enhanced chemiluminescence using the Chemi Doc system and related software (Hercules, CA, USA).

### Immunoassay for 8-OHdG

A competitive ELISA for 8-OHdG was performed using a DNA/RNA Oxidative Damage ELISA kit (Cayman Chemical Company, Ann Arbor, Michigan, USA) according to the manufacturer’s protocol. Serum samples were analyzed in duplicate. Standard 8-OHdG was assayed over a concentration range of 10.3–3,000 pg/mL in duplicate for each experiment.

### Total RNA Isolation From vWAT and RT-qPCR

vWAT tissue samples were homogenized using a polytron in an appropriate volume of QIAzol lysis buffer (Qiagen, Hilden, Germany). RNA was extracted by the miRNeasy micro kit (Qiagen). Total RNA (1 µg) was used to synthesize cDNA strands in a 20-µL-reaction volume using the SuperScript IV reverse Transcriptase for RT-PCR (Invitrogen). 50µM of random hexamers, 10mM of dNTP mix and 1 µg of total RNA were combined and heated at 65°C for 5 minutes and then incubate on ice for at least 1 minute. Annealed RNA was combined with RT reaction mix and incubated at 23°C for 10 minutes, 50-55°C for 10 minutes and 80°C for 10 minutes. Real-Time quantitative RT-PCR (QRT-PCR) was carried out with 50 nM gene-specific primers, IQ SYBR Green supermix (Bio-Rad), and cDNA samples (2µl) in a total volume of 25 µl. A melting curve analysis was completed following amplification from 55 to 95°C to assure product identification and homogeneity. The mRNA expression levels were repeated in triplicate and were normalized to a reference gene (*B*-actin, stable under our experimental conditions) by using the 2^-ΔCt^ method. PCR primers were designed by using the Primer 3 program (48), and synthesized and verified by sequencing at Eurofins Genomics (Ebersberg, Germany).

Primers used were as follows:

B-ACTIN forward: 5’-GCTACAGCTTCACCACCACA-3’
B-ACTIN reverse: 5’-AGGGCAACATAGCACAGCTT-3’
IL-1B forward: 5’-CACCTCTCAAGCAGAGCACAG-3’
IL-1B reverse: 5’-GGGTTCCATGGTGAAGTCAAC-3’
IL-10 forward: 5’-GTCATCGATTTCTCCCCTGTGA-3’
IL-10 reverse: 5’-TTCATGGCCTTGTAGACACCTTT-3’
PIK3R2 forward: 5’-ACCCGTGTAATTGGACATAGGA-3’
PIK3R2 reverse: 5’-GCTTCCATTTTTACTTTCTCTTCCA-3’
SPRED-1 forward: 5’-CGAGATGGGCACTAGGCTT -3’
SPRED-1 reverse: 5’-ACTCGTGGCGGTAGTGATTG-3’
MCP-1 forward: 5’-TGTCTCAGCCAGATGCAGTTAAT-3’
MCP-1 reverse: 5’- CATCTTGCCAGTGAATGAGTAGC-3’
F4/80 forward: 5’- TGCAGTTCAGAACCACAACACCTAC-3
F4/80 reverse: 5’- CCCGCAATGATAGCGCAAG-3’
CD206 forward: 5’-TCGGGTGAACGGAATGATTG-3’
CD206 reverse: 5’- AAGAGCCCTTGGGTTGAGGA-3’
CD11C forward: 5’- GGCTGAAATCACTTTCGACACA-3’
CD11C reverse: 5’-GAAGATGGGCTCATAGACCACGTA-3’
CD301 forward: 5’-AAGGCACACCTAGGCCACTG-3’
CD301 reverse: 5’-CTTCAGGTCTTGCACCAGCTG-3’
CD45 forward: 5’-CCGTTGTACACCAGAGATGA-3’
CD45 reverse: 5’-TCCCAAAATCAGTCTGCAC-3’
CD3 forward: 5’-GTCATTGCCACTCTGCTCCT-3’
CD3 reverse: 5’-CCAGACAGCCTTCCAGTCTC-3’
FOXP3 forward: 5’-CGGGAGAGTTTCTCAAGCAC-3’
FOXP3 reverse: 5’-CACAGGTGGAGCTTTTGTCA-3’
IL-13 forward: 5’-ATCGAGGAGCTGAGCAACAT-3’
IL-13 reverse: 5’-ATCCGAGGCCTTTTGGTTAC-3’
IL-4 forward: 5’-TCTCACGTCACTGACTGTA-3’
IL-4 reverse: 5’-CTTTCAG TGTTGTGAGCGT-3’
CCL5 forward: 5’-GCCCTCGCTGTCATCCTCAT-3’
CCL5 reverse: 5’-TGTACTCCCGAACCCATTTC-3’
CCL7 forward: 5′- GGCCTCCTCAACCCACTTCT-3′
CCL7 reverse: 5′−CCCTGGGAAGCTGTTATCTTCA-3′,
CCR2 forward: 5′-GTTCTCTTCCTGACCACCTTC-3′
CCR2 reverse: 5′-CTTCGGAACTTCTCACCAACA-3′
CCR5 forward: 5′-GGACTGAATAATTGCAGTAGTTC-3′
CCR5 reverse: 5′-TGTTTTCGGAAGAACACAGAG-3′
CHI313 forward: 5’-AGTACCCTATGCCGTTCAGG-3’
CHI313 reverse: 5’-CAGACCATTGCACCTCCTAA-3’
IL-4RA forward: 5’-TCATGGATGACGTGGTCAGT-3’
IL-4RA reverse: 5’-GTGTCGGAGACATTGGTGTG-3’

### miRNA and Total RNA Isolation From Serum and RT-qPCR

Total RNA, including small RNAs, were isolated from 100 uL of serum using Qiazol extraction method followed by column purification with a miRNeasy Mini kit (Qiagen) in accordance with the manufacturer’s protocol. 400 µL of Qiazol and 80µL of chloroform were added to 100µL of serum, followed by centrifugation for 15min. at 12,000×g at 4°C. 300µL of the RNA containing aqueous phase was transferred into a new tube, RNA was precipitated with 450uL of 100% (v/v) ethanol and loaded on miRNeasy purification columns. 700 µL of the sample were pipetted into an RNeasy MinElute spin column and centrifuged for 15 s at ≥ 8000 x g at room temperature. 700 µl of Buffer RWT were added to the column and centrifuge for 15 s at ≥ 8000 x g to wash the column and discard the flow-through. 500 µl of buffer RPE were loaded into columns and was centrifuged for 15 s at ≥ 8000 x g. The flow-through was discarded. After the centrifugation the RNeasy MinElute spin column were placed into a new tube and was centrifuged for 5 min at ≥8000 x g. Purified RNA was eluted from the column matrix with 20 µL of RNase free water. miRNA and total RNA yield was quantified using a Nanodrop1000 device (Thermofisher, USA). MicroRNA-126 (miR126) was quantified along with RNU6B (reference transcript) by RT-qPCR with TaqMan^®^ miRNA assays from Applied Biosystems, according to the manufacturer’s protocol. Its expression levels were normalized to a reference gene (RNU6B) by using the 2^−ΔCt^ method. The analyses were performed on five independent experiments, each in triplicate. For total RNA, 1 µg of RNA from serum sample was used to synthesize cDNA. Serum expression levels of PIK3R2 and SPRED1 were determined by RT-QPCR. Each sample was assayed in triplicate and was normalized to the GAPDH gene. PCR primers were designed by using the Primer 3 program (48), and synthesized and verified by sequencing at Eurofins Genomics (Ebersberg, Germany).

Primers used were as follows:

GAPDH forward: 5’-GCACCGTCAAGGCTGAGAAC-3’
GAPDH reverse: 5’-TGGTGAAGACGCCAGTGGA-3’
PIK3R2 forward: 5’-ACCCGTGTAATTGGACATAGGA-3’
PIK3R2 reverse: 5’-GCTTCCATTTTTACTTTCTCTTCCA-3’
SPRED-1 forward: 5’-CGAGATGGGCACTAGGCTT -3’
SPRED-1 reverse: 5’-ACTCGTGGCGGTAGTGATTG-3’

### Statistical Analysis

All results were analyzed with the GraphPad Prism 6 software system (LaJolla, CA, USA). Data are expressed as the mean ± SEM and are normally distributed. The statistical significance of the differences between experimental groups was determined using one-way analysis of variance (ANOVA), followed by the “Sidak correction for Multiple Comparison”. Differences were considered statistically significant at p < 0.05.

## Results

### Administration of T2 to HFD Rats Reduces Adiposity, Cholesterol and Glucose Serum Levels and Increases Resting Metabolic Rate Without Inducing a Thyrotoxic State

**Figure 1:**
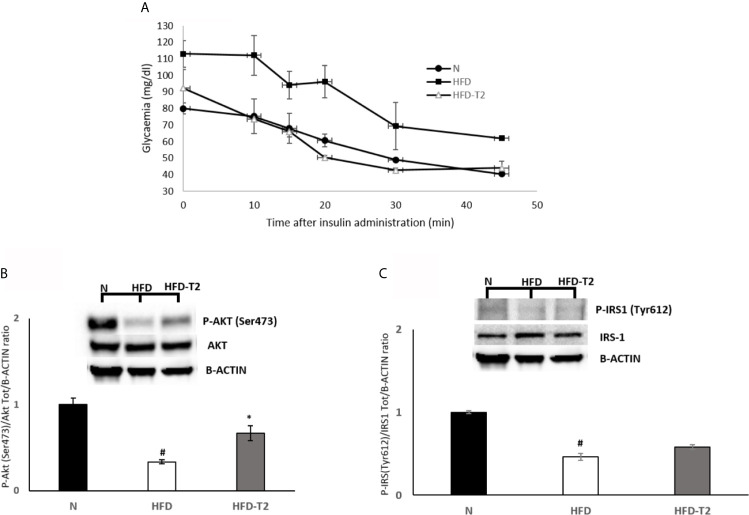
Effects of T2 on insulin responsiveness. **(A)** Insulin Tolerance test (ITT). Glycemia after insulin administration at different time points in N, HFD, and HFD + T2 rats. Values are means ± SEM of five independent experiments (n = 5). **(B, C)** Western blot images and densitometry showing relative expression of two proteins involved in insulin signaling in vWAT. **(B)** Phospho AKT (Ser473) **(C)** Phospho IRS-1 (Tyr612); B-actin has been used as a loading control. Representative blots are shown. The histograms represent means ± SEM of five independent experiments (n = 5), *P < 0.05 *vs.* HFD, ^#^P < 0.05 *vs.* N. N: rats receiving a standard diet for 14 weeks; HFD: rats receiving a HFD for 14 weeks; HFD-T2: rats receiving a HFD for 14 weeks with a daily administration of T2 during the last 4 weeks.

At 10 weeks, rats fed on a HFD gained significantly more weight when compared to animals fed on a standard diet (417.25 *vs* 357.75, respectively). After 10 weeks of HFD, the simultaneous administration of T2 for further 4 weeks of diet is effective in counteracting the further increase in adiposity. The HFD rats after 14 weeks of HFD gained about 63% more weight than N rats (+195.7 g *vs*. +124.0 g respectively) (**Table 1**). Conversely, the HFD-T2, showed, even not significant, a fair amount of less weight gain in the last 4 weeks, with a value of 179.8 g *vs*. 195.7 g of HFD group, corresponding to −8% (**Table 1**). During the last 4 weeks of treatment (when T2 administration to a subgroup of HFD rats begins), there is no remarkable increase in body weight in HFD rats *vs* N group (+9.0 g *vs.* +8.0 g, respectively). We do not observe changes as the growth curve of rats stabilizes. Instead, despite this in HFD-T2 rats the body weight gain is significantly reduced when compared to HFD animals (+4.8 g *vs*. +9.0 g, respectively) (**Table 1**). T2 administration significantly increased the resting metabolic rate when compared to HFD rats, without affecting energy intake (**Table 1**). The decreased in Respiratory Quotient observed in HFD-T2 rats, like that of HFD animals, indicates a shift in metabolism toward utilization of lipids (**Table 1**). Furthermore, T2 was able to counteract the increase both in cholesterol serum levels and in glycaemia. In fact, in HFD-T2 rats, the serum cholesterol levels were reduced by ~23% compared to HFD rats and glycaemia was reduced by ~15% (not significant) compared to HFD group (**Table 1**). Collectively, these metabolic parameters are indicative of an increased burning of fat upon T2 administration to long-lasting HFD fed rats. These effects are not associated with a thyrotoxic state. Indeed, TSH serum levels, free T3 serum levels, and free T4 serum levels did not significantly change from the normal euthyroid values (**Table 1**). The insulin tolerance test revealed that T2 was able to reverse the HFD-induced impaired insulin responsiveness (**Figure 1A**). In vWAT of HFD rats the phosphorylation levels of Protein Kinase B (Akt/PKB) and Insulin Receptor Substrate (IRS-1), two proteins involved in downstream insulin signaling, were significantly reduced when compared to N animals (**Figures 1B, C**). The administration of T2 significantly enhanced phosphorylation of Akt compared to HFD group (**Figure 1B**), while the phosphorylation of IRS-1 was increased by about ~18% (**Figure 1C**).

**Table 1:** Body weight gain, energy intake, resting metabolic rate, respiratory quotient, serum cholesterol levels, glycaemia and serum TSH, FT3, FT4 in N, HFD and HFD-T2 rats.

Parameters	N	HFD	HFD-T2
**Body weight gain (during 14 weeks)**	124.0 ± 10.5	195.7 ± 7.1	179.8 ± 8.4
**Body weight gain (during the last 4 weeks)**	8.0 ± 2.5	9.0 ± 1.0	4.8 ± 1.9*
**Energy Intake (kJ)**	5118 ± 408	6866 ± 504^#^	7042 ± 363^#^
**Resting Metabolic Rate (LtO2/h/Kg0.75)**	0.90 ± 0.015	0.74 ± 0.009^#^	0.89 ± 0.034*
**Respiratory Quotient**	0.97 ± 0.063	0.76 ± 0.013^#^	0.74 ± 0.008^#^
**Cholesterol (mg/dL)**	41.3 ± 2.42	77.1 ± 2.64^#^	55.0 ± 4.03**
**Glycaemia (mg/dL)**	81.3 ± 3.38	110.1 ± 10.14^#^	93.2 ± 4.51
**TSH (μU/mL)**	0.0068 ± 0.0003	0.0071 ± 0.0013	0.0066 ± 0.0005
**Free T_3_ (pg/mL)**	1.85 ± 0.2	1.97 ± 0.3	1.80 ± 0.2
**Free T4 (ng/mL)**	0.91 ± 0.05	0.89 ± 0.06	0.82 ± 0.07

Values are means ± SEM of five independent experiments (n = 5), ^#^P < 0.05 vs. N; *P < 0.05 vs. HFD; **P < 0.05 vs. N and HFD. N: rats receiving a standard diet for 14 weeks; HFD: rats receiving a HFD for 14 weeks; HFD-T2: rats receiving a HFD for 14 weeks with a daily administration of T2 for the last 4 weeks.

**Figure 2:**
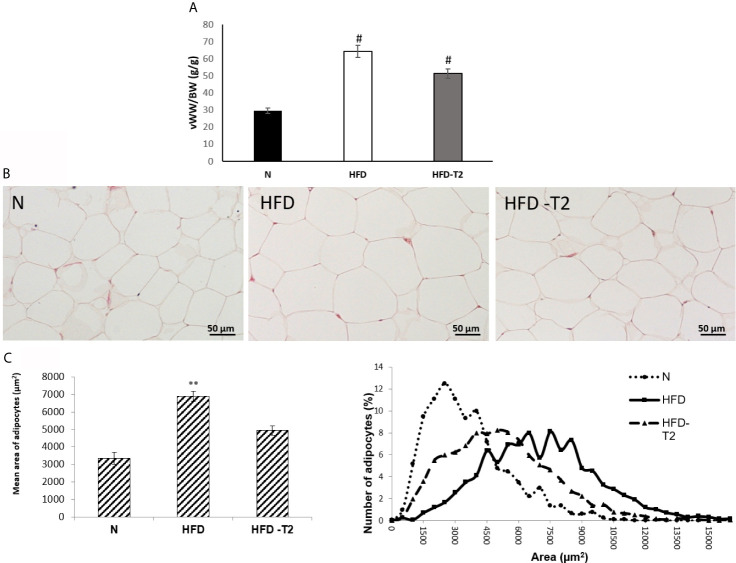
Effects of T2 administration on visceral fat accumulation and on vWAT morphology. **(A)** Ratio of visceral white adipose tissue weight (vWW)/body weight (BW); **(B)** histological and **(C)** morphometrical analysis of visceral white adipocyte size. Representative staining of five independent treatments are shown and were reproduced. Values are means ± SEM of five independent experiments (n = 5), ^#^P < 0.05 *vs*. N; **P < 0.05 *vs.* N and HFD –T2. N: rats receiving a standard diet for 14 weeks; HFD: rats receiving a HFD for 14 weeks; HFD-T2: rats receiving a HFD for 14 weeks with a daily administration of T2 during the last 4 weeks.

As reported in **Figure 2**, the ratio of vWAT weight (mg)/body weight (g) was reduced by about 27% in HFD-T2 when compared to that of HFD rats (**Figure 2A**).

Moreover, while in HFD rats, the adipocyte size was significantly increased with respect to standard diet-fed (N) controls (**Figures 2B, C**), after T2 administration, the adipocytes size was significantly reduced in comparison to HFD rats (**Figures 2B, C**).

### Administration of T2 to HFD Rats Reduces the Macrophage Infiltration, Induces a Phenotypic Switching From M1 to M2 Macrophages and Affects Lymphocytes in vWAT

**Figure 3:**
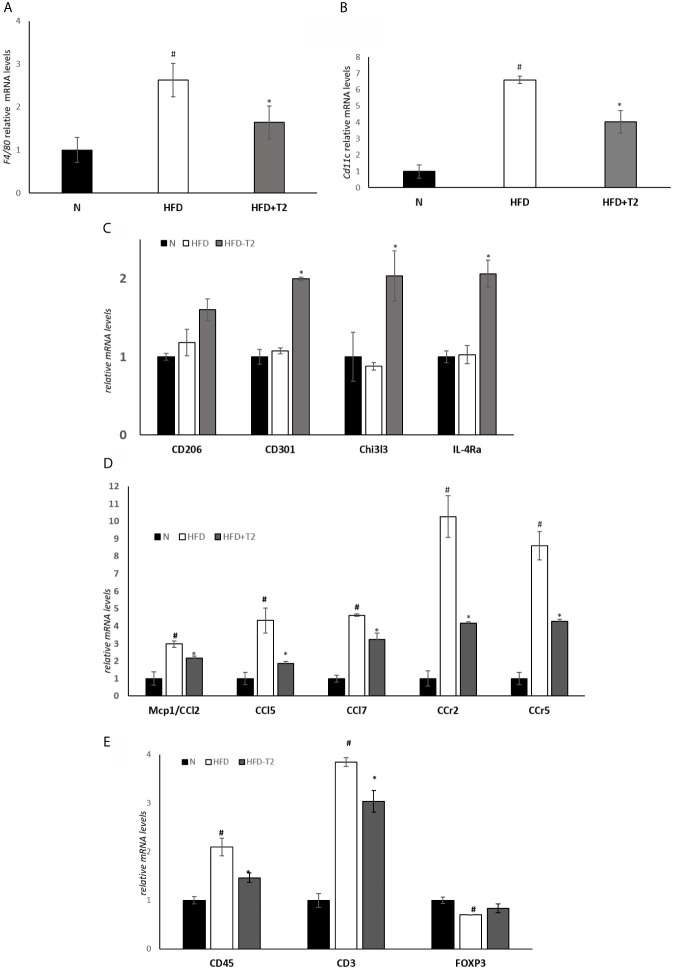
Effect of T2 administration on expression of markers of macrophage infiltration and adipose tissue lymphocytes in vWAT. RT-qPCR analysis of **(A)** F4/80, **(B)** CD11C and **(C)** CD206, CD301, Chi3l3, IL-4Ra, **(D)** Mcp1/CCl2, CCl5, CCl7, CCr2, CCr, **(E)** CD45, CD3, FOXP3 in vWAT of N, HFD, and HFD-T2 rats. The histograms represent means ± SEM of five independent experiments (n = 5), ^#^P < 0.05 *vs.* N; *P < 0.05 *vs.* HFD. N: rats receiving a standard diet for 14 weeks; HFD: rats receiving a HFD for 14 weeks; HFD-T2: rats receiving a HFD for 14 weeks with a daily administration of T2 during the last 4 weeks.

We explored the expression of markers related to HFD-induced macrophage infiltration leading to chronic low-grade inflammation. Visceral resident macrophage abundance was assessed by detecting the expression levels of F4/80, a known macrophage quantitative marker (49). The expression levels of F4/80 were increased in the HFD rats compared with the N animals and significantly reduced in HFD-T2 group compared with HFD rats (**Figure 3A**). The expression levels of the pro-inflammatory M1 macrophage marker CD11C were increased in HFD rats compared with N rats. T2 significantly reversed CD11C gene expression levels (**Figure 3B**). Moreover, the expression levels of anti-inflammatory M2 macrophage markers such as CD206, CD301, Chitinase 3-like 3 (Chi3l3) and Interleukin 4 receptor alpha (IL-4Ra) were unchanged in HFD animals compared with N group. T2 administration to HFD animals significantly increased CD301, Chi3l3 and IL-4Ra gene expression levels when compared to HFD rats (**Figure 3C**) and enhanced CD206 gene expression levels (about 36%). The expression levels of several chemokine as Mcp1/Ccl2, Chemokine (C-C motif) ligand 5 (Ccl5), Chemokine (C-C motif) ligand 7 (Ccl7) and of their receptors, C-C chemokine receptor type 2 (Ccr2) and C-C chemokine receptor type 5 (Ccr5), were significantly incresed in HFD rats compared with N animals (**Figure 3D**), indicating that the increased macrophages infiltration in HFD condition could also depend on chemokine. T2 administration to HFD rats was able to significantly reduce the expression levels of all the measured chemokines and of their receptors (**Figure 3D**). Furthermore, in HFD rats the expression levels of CD45 and CD3, lymphocytes markers, were significantly incresed when compared to N animals (**Figure 3E**). In T2-treated rats, instead, the expression levels of both CD45 and CD3 were significantly reduced (**Figure 3E**). The expression levels of Forkhead box P3 (Foxp3), involved in anti-inflammatory signals in adipose tissue (50), were significantly decrease in HFD rats when compared to N animals (**Figure 3E**) while T2 treatment induced an increase by about ~18% (not significant) (**Figure 3E**). These results indicate that T2 in HFD rats reduces macrophages infiltration, regulates their polarization in vWAT toward an anti-inflammatory phenotype and reduces lymphocytes recruitment.

### Administration of T2 to HFD Rats Modulates Serum Adiponectin and the Expression of Anti-Inflammatory and Pro-Inflammatory Cytokines

**Figure 4:**
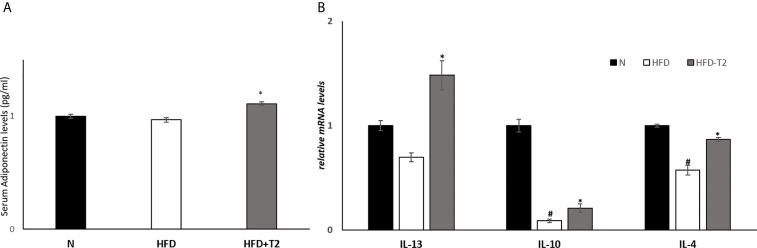
Effects of T2 administration on adiponectinserum levels and on expression of IL-13, IL-10 and IL-4 in vWAT. **(A)** Adiponectin was detected by a competitive Enzyme Linked-Immunosorbent Assay (ELISA) in serum of N, HFD, and HFD-T2 rats; **(B)** RT-qPCR analysis of IL-13, IL-10, IL-4 in vWAT of N, HFD, and HFD-T2 rats. Values are means ± SEM of five independent experiments (n = 5), *P < 0.05 *vs.* HFD; ^#^P < 0.05 vs. N. N: rats receiving a standard diet for 14 weeks; HFD: rats receiving a HFD for 14 weeks; HFD-T2: rats receiving a HFD for 14 weeks with a daily administration of T2 during the last 4 weeks.

We measured the serum levels of adiponectin and mRNA levels of interleukin-10 (IL-10), interleukin-4 (IL-4) and interleukin-13 (IL-13) in vWAT, agents with anti-inflammatory effects (51–53). Serum adiponectin levels were significantly increased in T2-treated rats compared to HFD animals (**Figure 4A**). The expression levels of IL-10, a cytokine whose production is also induced by adiponectin (54), as well as that of IL-4 and IL-13 were significantly reduced in HFD rats compared to N controls (**Figure 4B**), while T2 administration to HFD rats significantly increased their expression compared to those found in HFD rats (**Figure 4B**).

**Figure 5:**
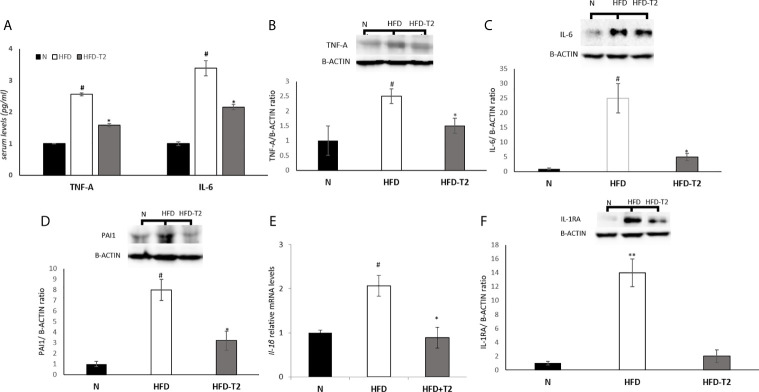
Effects of T2 administration on TNF-A and IL-6 serum levels and on expression of some pro-inflammatory cytokines in vWAT. **(A)** TNF-A and IL-6 were detected by a competitive Enzyme Linked-Immunosorbent Assay (ELISA) in serum of N, HFD, HFD-T2 rats; **(B–D, F)** Western blot images and densitometry showing relative expression of the main pro-inflammatory cytokines in vWAT. **(B)** Tumor necrosis factor A (TNF-A); **(C)** Interleukin-6 (IL-6), **(D)** Plasminogen Activator Inhibitor 1 (PAI1); **(F)** Interleukin-1 Receptor Antagonist (IL-1RA); B-ACTIN has been used as a loading control. Representative blots are shown; **(E)** RT-qPCR analysis of IL-1B in vWAT. The histograms represent means ± SEM of five independent experiments (n = 5), *P < 0.05 *vs.* HFD, ^#^P < 0.05 *vs.* N, **P < 0,05 *vs.* N and HFD-T2. N: rats receiving a standard diet for 14 weeks; HFD: rats receiving a HFD for 14 weeks; HFD-T2: rats receiving a HFD for 14 weeks with a daily administration of T2 during the last 4 weeks.

Next, we measured the serum levels of two pro-inflammatory cytokines, TNF-A and IL-6, and the vWAT protein levels of several pro-inflammatory cytokines including TNF-A, IL-6 and PAI-1 as well as the mRNA levels of IL-1B. As reported in **Figure 5**, serum and tissue protein levels of TNF-A and IL-6 resulted significantly enhanced in HFD rats when compared to N group, while were significantly reduced in T2-treated animals (**Figures 5A–C**), indicating that T2 was able to reduce both the production and the secretion of these cytokines. The protein levels of PAI-1 and the mRNA levels of Interleukin 1 beta (IL-1B) were also increased in HFD rats compared with N rats (**Figures 5D, E**). T2 administration to HFD rats significantly decreased PAI-1 and IL-1B levels (**Figures 5D, E**). Moreover, the protein level of Interleukin-1 Receptor Antagonist (IL-1RA) was significantly decreased in HFD-T2 rats compared to the HFD ones (**Figure 5F**). These results indicate that T2 reverse pro-inflammatory status induced by HFD.

### Administration of T2 to HFD Rats Modulates Serum Levels of Irisin and Its Downstream AMPK/SIRT1 Pathway in vWAT

**Figure 6:**
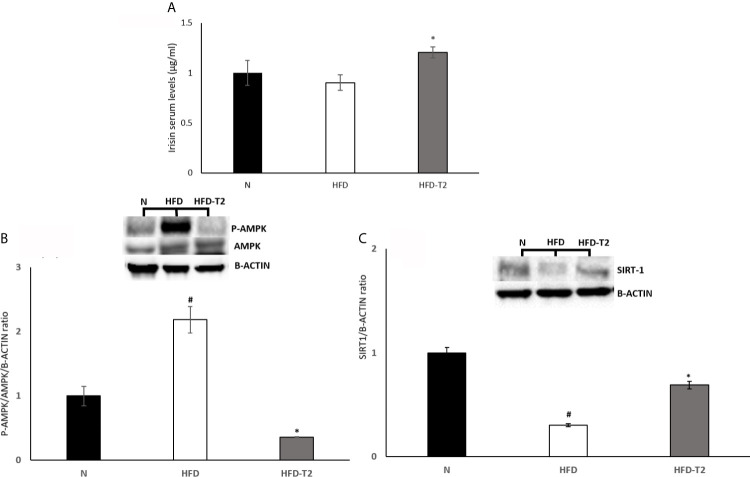
Effects of T2 administration on Irisin serum levels and on P-AMPK and SIRT-1 expression in vWAT. **(A)** Irisin was detected by a competitive Enzyme Linked-Immunosorbent Assay (ELISA) in serum of N, HFD, HFD-T2 rats. The histograms represent means ± SEM of five independent experiments (n=5), *P < 0.05 *vs.* HFD. N: rats receiving a standard diet for 14 weeks; HFD: rats receiving a HFD for 14 weeks; HFD-T2: rats receiving a HFD for 14 weeks with a daily administration of T2 during the last 4 weeks. **(B, C)** Western blot images and densitometry showing relative expression of P-AMPK and SIRT-1. The histograms represent means ± SEM of five independent experiments (n = 5), ^#^P < 0.05 *vs.* N; *p < 0.05 *vs.* HFD. N: rats receiving a standard diet for 14 weeks; HFD: rats receiving a HFD for 14 weeks; HFD-T2: rats receiving a HFD for 14 weeks with a daily administration of T2 during the last 4 weeks.

Subsequently, we measured the serum levels of irisin, which treatment it has been reported to suppress expression of pro-inflammatory cytokines (55, 56). As reported in **Figure 6A**, serum levels of this miokine were significantly increased by T2 treatment (**Figure 6A**). Furthermore, it has been shown that irisin attenuates adipose tissue inflammation *via* 5’ AMP-activated protein kinase (AMPK)/Sirtuin 1 (SIRT-1) pathway (57–59). For this reason, we measured the AMPK phosphorylation levels and SIRT-1 protein content. The results showed that, in HFD rats, the phosphorylation of AMPK was significantly increased when compared to N group (**Figure 6B**). In T2-treated rats, AMPK phosphorylation levels were significantly reduced compared to HFD animals (**Figure 6B**). Instead, SIRT-1 protein levels were reduced in HFD rats compared to N group (**Figure 6C**) and increased in T2-treated rats when compared to HFD animals (**Figure 6C**). These results indicate that SIRT-1 activation by irisin could be a mechanism by which T2 affects the inflammatory state.

### Administration of T2 to HFD Rats Ameliorates Adipose Tissue Hypoxia

**Figure 7:**
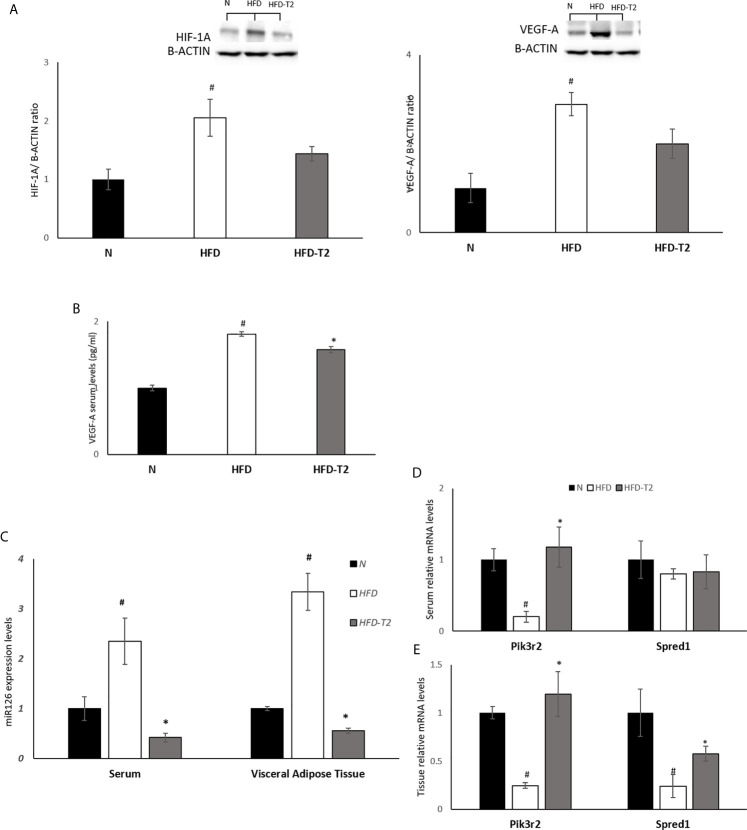
Effects of T2 administration on HIF-1A and VEGF-A expression in vWAT and on miR126 and of its targets PIK3R2 and SPRED1 in serum and vWAT. **(A)** Western blot image and densitometry showing relative expression of HIF-1A and VEGF-A in vWAT. Representative blots are shown; **(B)** VEGF-A was detected by a competitive Enzyme Linked-Immunosorbent Assay (ELISA) in serum of N, HFD, HFD-T2 rats. **(C)** qPCR analysis of miR126 in serum and vWAT of N, HFD, and HFD-T2 rats; **(D)** qPCR analysis of PIK3R2 and SPRED1 in serum of N, HFD, and HFD-T2 rats; **(E)** qPCR analysis of PIK3R2 and SPRED1 in vWAT of N, HFD, and HFD-T2 rats. The histograms represent means ± SEM of five independent experiments (n = 5), ^#^P < 0.05 *vs.* N; * p < 0.05 *vs.* HFD. N: rats receiving a standard diet for 14 weeks; HFD: rats receiving a HFD for 14 weeks; HFD-T2: rats receiving a HFD for 14 weeks with a daily administration of T2 during the last 4 weeks.

Then, we evaluated the effect of T2 on the hypoxic environment induced by the hyperlipidic diet. In HFD rats, the protein levels of HIF-1A and of its target VEGF-A were significantly increased, as expected (**Figure 7A**). In T2-treated rats the levels of HIF-1A and VEGF-A were decreased by about 30% *vs.* HFD but did not reach statistical significance. In HFD-T2 rats, instead, the serum levels of VEGF-A were significantly reduced when compared to HFD animals (**Figure 7B**). These data suggest a putative role of T2 in reducing vWAT hypoxia. As hypoxia stimulates the expression of genes that act to increase oxygen availability by decreasing oxygen consumption and stimulating angiogenesis, the next step was to evaluate whether T2 could counteract HFD-induced hypoxia by modulating angiogenesis *via* the endothelial cell-specific miR126 that plays an essential role in angiogenesis (23, 60). In HFD rats, serum and vWAT levels of miR126 were significantly increased (**Figure 7C**) while, in T2-treated animals, its expression was drastically reduced (**Figure 7C**). Consistently, we found that, compared to HFD animals, T2 administration significantly enhanced serum and tissue (vWAT) levels of the miR126 target PIK3R2 (**Figures 7D, E**). In addition, in T2-treated animals, the expression levels of SPRED1, while unchanged in serum, were significantly increased at the tissue level (**Figures 7D, E**).

### Administration of T2 to HFD Rats Reduces the Serum Levels of 8-OHdG

**Figure 8:**
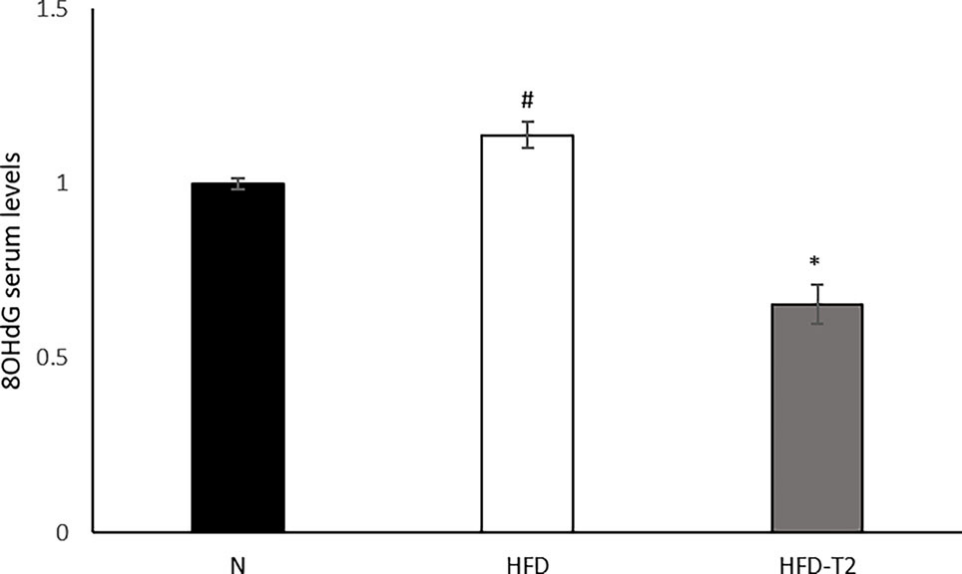
Effects of T2 administration on 8-OHdG serum levels. 8-OHdG was detected by a competitive Enzyme Linked-Immunosorbent Assay (ELISA) in serum of N, HFD, HFD-T2 rats. The histograms represent means ± SEM of five independent experiments (n = 5), *P < 0.05 *vs.* HFD and ^#^P < 0.05 *vs.* N. N: rats receiving a standard diet for 14 weeks; HFD: rats receiving a HFD for 14 weeks; HFD-T2: rats receiving a HFD for 14 weeks with a daily administration of T2 during the last 4 weeks.

Finally, we measured the serum levels of 8-OHdG, an oxidized derivative of the guanine base used as a marker of systemic oxidative stress (61). As shown in **Figure 8**, serum levels of 8-OHdG increased in HFD rats and were significantly reduced in T2 treated ones, again highlighting and confirming a protective role of T2 against the oxidative damage (46) induced by a hyperlipidic diet.

## Discussion

Adipose tissue is an important metabolic organ with a plethora of physiological functions. Lipids accumulation and changes in adipocyte size lead to chronic inflammation (62). Diets containing a high concentration of fats may induce overweight and obesity. In such conditions, adipocytes increase in size with consequent increase in intercapillary distance and decrease in oxygen tension that activates the transcription factor HIF-1A and generates several adverse effects for the adipocyte, among which: i) cell death; ii) inhibition of production of adiponectin; iii) production and release of inflammatory cytokines (63), strong predictor of developing insulin resistance (64), and iv) increased oxidative stress which is able to damage DNA. These conditions, combined with the induction of unhealthy angiogenesis, stimulates inflammation (6, 12). The first step of this study was to treat the animals with a long-lasting HFD diet able to determine a condition of overweight as well as of an inflammatory state. In these animals, we showed, in vWAT, both an increase in fat mass and in adipocyte size. Administration of T2 to HFD rats reduced the adiposity, stimulated the resting metabolic rate (**Table 1**) and improved insulin responsiveness (**Figure 1**). T2 ameliorated the insulin sensitivity of the vWAT by increasing Akt and IRS-1 phosphorylation levels.

The HFD-induced alterations of adiposity were associated with a substantial inflammatory state highlighted by an increase in macrophage infiltration, an increase of pro-inflammatory cytokines levels and induction of hypoxia. Many studies have reported that adipocyte death is the main stimulus that regulates macrophage infiltration in adipose tissue (65, 66). Indeed, numerous macrophages are found in close proximity of dead adipocytes (66, 67). They fuse creating the so-called “crown-like” structures. The “crown-like” structures express F4/80 (a well-characterized and extensively referenced mouse macrophage marker) (49) and pro-inflammatory cytokines which prove the presence of M1 macrophages and indicate inflammation (18, 66). Our data show that T2 administration to HFD rats decreases the expression levels of F4/80 and the expression levels of several chemokines (Mcp1/Ccl2, Ccl5, Ccl7) and their receptors (Ccr2, Ccr5), suggesting a reduction in macrophage infiltration in vWAT of HFD-T2 rats. In addition, the decrease of CD11C and the increase of CD206, CD301, Chi3l3 and IL-4Ra expression levels in HFD-T2 rats indicate that T2 is able to influence macrophages polarization inducing, in vWAT, a phenotypic switching from M1 pro-inflammatory macrophage to M2 anti-inflammatory macrophage. Furthermore, the reduction in the CD45 and CD3 expression levels and the increase of Foxp3 highlight that T2 reduces lymphocytes recruitment.

Serum levels of adiponectin, an adipokine with anti-inflammatory function (68), were significantly increased in HFD-T2 *vs*. HFD animals. However, in HFD animals the levels of adiponectin did not significantly differ from those of N controls (only a decrease of about 4% was observed). The observed lack of effects of HFD on serum adiponectin levels could be explained as follows: a) the treatment was not long enough. In fact, it has been shown that the expression of adiponectin was reduced after at least 20 weeks of HFD (69); b) the diet composition in fatty acids elicited an interference (satured/monounsatured/polyunsatured) (70, 71); c) diet-induced overweight/metabolic syndrome is not always associated with decreased adiponectin levels in rats (72).

In HFD-T2 rats, the increase in serum levels of adiponectin was associated with an increase in the expression levels of the anti-inflammatory cytokine, IL-10. This cytokine has insulin-sensitizing and anti-inflammatory properties by antagonizing TNF-A and IL-6 (73). Furthermore, the expression of IL-10 has been reported to be reduced in obesity, metabolic syndrome and type 2 diabetes. These results are in line with the insulin-sensitizing effects of T2. In addition, in HFD-T2 rats, the expression levels of IL-4 and IL-13, two cytokines with anti-inflammatory action, resulted to be increased. Our data also showed that T2 administration is able to reduce serum and tissue protein levels of pro-inflammatory cytokines such as TNF-A and IL-6, vWAT protein levels of PAI-1, and the expression levels of IL-1B, all factors strongly increased in HFD. The protein levels of IL-1RA, an acute-phase protein, were also significantly downregulated in the HFD-induced inflammatory state.

Then, we investigated the role of irisin in the anti-inflammatory action exerted by T2. Recently, we have shown that T2 induces the browning of the subcutaneous white adipose tissue (44) acting through irisin, among others pathways. Furthermore, it has been reported that irisin treatment suppresses the expression of pro-inflammatory cytokines (56, 74), induces the phenotypic switching of adipose tissue macrophages from M1 to M2 state (75) and attenuates adipose tissue inflammation *via* AMPK/SIRT1 pathway (57, 58). Our results showed that in HFD-T2 rats, the serum levels of irisin were significantly increased highlighting that irisin could be a mechanism of action by which T2 affects inflammatory state. Moreover, T2 administration reduced AMPK phosphorylation and increased SIRT1 protein levels, indicating a likely activation of an irisin-SIRT1 pathway in T2-treated animals.

The inflammatory state of HFD rats was also associated to an increase in protein levels of HIF-1A and of its target, VEGF-A. T2 administration reduced the levels of both these factors by about 30% and of serum VEGF-A indicating that T2, through the decrease of the fat mass, can positively affect the hypoxic environment within vWAT. Accordingly, morphological/morphometrical analysis showed, in HFD-T2 animals, a reduced adipocyte size when compared to HFD ones. It is known that, as a consequence of hypoxia, vWAT undergoes an unhealthy increase of angiogenesis. Moreover, tissue hypoxia together with the induction of angiogenesis stimulates inflammation (6, 76, 77). As mentioned above, recent evidences highlighted a role of miRNAs in angiogenesis. Among the others, miR126 controls angiogenesis and regulates the formation and maintenance of new vessels (21) by targeting 3’UTR mRNAs of SPRED1 and PIK3R2, both negative regulators of the VEGF pathway (23, 60). Here, we report, for the first time, that the administration of T2, through the regulation of miR126, can preserve the physiological angiogenesis and the vascular integrity in condition of increased fat mass and inflammation by regulating the above-mentioned genes.

**Figure 9:**
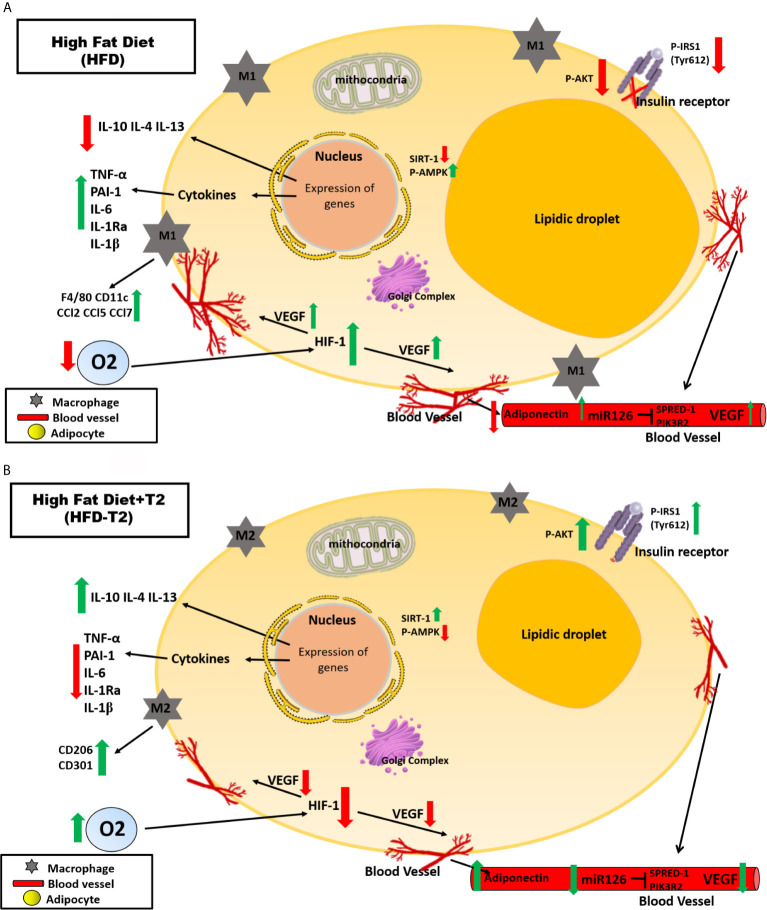
Schematic representation of the effects exerted on adipocytes in vWAT by A) High Fat Diet (HFD) for 14 weeks and B) by High Fat Diet for 14 weeks and T2 administered daily during the last 4 weeks (HFD-T2). In vWAT from overweight rats, hypoxia induces the synthesis of several angiogenic factors (e.g.,VEGF-A) and the expression of inflammatory cytokines (e.g., TNF-A, IL-6) while decreasing the expression of adiponectin. In this way, a vicious circle is established in which the activation of angiogenesis first determines a further increase in adipocyte volume and, subsequently, an increase in the inflammatory state of the adipose tissue. In overweight rats treated with T2, the inflammatory state is reverted. The diiodothyronine is able to reduce the expression of inflammatory cytokines and the macrophage infiltration producing at the same time a phenotypic switching from M1 pro-inflammatory to M2 anti-inflammatory macrophages. Moreover, while HFD is associated with increased levels of circulating miR126, T2 administration results in a significant down representation of such marker of angiogenesis. These findings highlight the ability of T2 to reverse the inflammatory state induced by the HFD.

A protective effect of T2 on DNA damage is furthermore brought to light since we show here that T2 is able to reduce systemic oxidative stress as highlighted, in HFD-T2 animals by the reduced serum levels of 8-OHdG. A schematic representation of what was described is illustrated in **Figure 9**.

The already described effect of T2 in stimulating liver fatty acid oxidation and an inefficient mitochondrial utilization of fatty acid substrates (78), together with the known effects exerted by the iodothyronine on the other metabolically active tissues (skeletal muscle and brown adipose tissue) (35, 40, 43) in rats receiving a HFD, may also play a role in the above described favorable effect on inflammation. In fact, we have shown that T2 ameliorates muscle glucose uptake by increasing the response to insulin (40),, and has lipolytic effects in the liver mediated by autophagy and increased fatty acid oxidation (79). Notably, T2 directly activates SIRT1, leading to the deacetylation of PGC1α and the activation of its transcriptional activity to induce expression of the genes involved in fatty acid oxidation (35). Furthermore, long-term treatment with T2 promotes visceral adipose lipolysis through HSL activation and effects on adipocyte morphology (already measurable after 2 weeks and persistent at 4 weeks of treatment), tissue vascularization and proteins involved in lipid storage and oxidative stress (47).

The data of the present study show, for the first time, that T2 is effective in counteracting inflammation and oxidative stress induced in rats by a long-lasting high fat diet. We focused the attention on adipose tissue but we do not exclude that other tissues may be involved. Moreover, further studies are needed to better evaluate/individuate other possible mechanisms underlying the aforementioned actions.

## Data Availability Statement

The original contributions presented in the study are included in the article/supplementary material. Further inquiries can be directed to the corresponding authors.

## Ethics Statement

The animal study was reviewed and approved by Committee on the Ethics of Animal Experiments of the University of Campania “L.Vanvitelli” (Italy) and the Italian Minister of Health (Permit Number: 704/2016-PR of the 15/07/2016).

## Author Contributions

Data curation, RS, FC, RM, and GP. Funding acquisition, FG and ALa. Investigation, RS, FC, and GP. Methodology, RS, FC, RM, and GP. Resources, FG and ALa. Supervision, PL, ES, ALo, MM, FG, and ALa. Writing—Original Draft, RS and FC. Writing—Review & Editing, RS, FC, RM, PL, ES, ALo, MM, FG, and ALa. All authors contributed to the article and approved the submitted version.

## Funding

This research was financially supported by a grant “Progetto di Ricerca di rilevante Interesse Nazionale (PRIN)” 2017, protocol 2017J92TM5_003.

## Conflict of Interest

The authors declare that the research was conducted in the absence of any commercial or financial relationships that could be construed as a potential conflict of interest.
